# Comparative Evaluation of Microbiota Dynamics and Metabolites Correlation Between Spontaneous and Inoculated Fermentations of Nanfeng Tangerine Wine

**DOI:** 10.3389/fmicb.2021.649978

**Published:** 2021-05-11

**Authors:** Xiangyu Qiu, Linlin Yu, Weiying Wang, Riming Yan, Zhibin Zhang, Huilin Yang, Du Zhu, Bo Zhu

**Affiliations:** ^1^Key Laboratory of Protection and Utilization of Subtropic Plant Resources of Jiangxi Province, College of Life Sciences, Jiangxi Normal University, Nanchang, China; ^2^Key Laboratory of Bioprocess Engineering of Jiangxi Province, College of Life Sciences, Jiangxi Science and Technology Normal University, Nanchang, China; ^3^College of Life Sciences, Gannan Normal University, Ganzhou, China

**Keywords:** tangerine wine, high-throughput sequencing, fermentation microbiota, metabolites, Non-Saccharomyces cerevisiae

## Abstract

Understanding the evolution of microorganisms and metabolites during wine fermentation is essential for controlling its production. The structural composition and functional capacity of the core microbiota determine the quality and quantity of fruit wine. Nanfeng tangerine wine fermentation involves a complex of various microorganisms and a wide variety of metabolites. However, the microbial succession and functional shift of the core microbiota in this product fermentation remain unclear. Therefore, high-throughput sequencing (HTS) and headspace-gas chromatography-mass spectrometry (HS/GC-MS) were employed to reveal the core functional microbiota for the production of volatile flavors during spontaneous fermentation (SF) and inoculated fermentation (IF) with *Saccharomyces cerevisiae* of Nanfeng tangerine wine. A total of 13 bacterial and 8 fungal genera were identified as the core microbiota; *Lactobacillus* and *Acetobacter* were the dominant bacteria in SF and IF, respectively. The main fungal genera in SF and IF were *Hanseniaspora*, *Pichia*, and *Saccharomyces* with a clear succession. In addition, the potential correlations analysis between microbiota succession and volatile flavor dynamics revealed that *Lactobacillus*, *Acetobacter*, *Hanseniaspora*, and *Saccharomyces* were the major contributors to the production of the volatile flavor of Nanfeng tangerine wine. The results of the present study provide insight into the effects of the core functional microbiota in Nanfeng tangerine wine and can be used to develop effective strategies for improving the quality of fruit wines.

## Introduction

Fruit wines are fermented alcoholic beverages made of fruits other than grapes, and they may also have additional flavors taken from other fruits, flowers, and herbs. The high demand for fruit wine, a derived product, has provided an opportunity for the development of value-added products. The fruit wine market is now progressing toward diversification by developing original, novel, and enriched products through innovative formulas, technologies, and alternative raw materials (Joshi et al., 2017). For a long time, citrus has been one of the most widely cultivated fruits worldwide, and abundant citrus varieties and resources are available in China (Liu R. et al., 2015). The flavor of citrus wine varies depending on the variety and processing method. Nanfeng tangerine, a unique citrus cultivar and a royal tribute in ancient China, has been cultivated in Nanfeng district (Jiangxi Province, China) for over 1,300 years. In recent decades, the yield and area cultivated for Nanfeng tangerine have increased to meet the strong market demand for this product, which contains various nutrients, such as amino acids, glucose, fructose, citric acid, vitamin C, vitamin B1, vitamin B2, calcium, phosphorus, iron, and other inorganic salts (Zhang et al., 2011). However, the characteristics of high sugar content and thin peel make it difficult to preserve at room temperature, and large amounts of rotten Nanfeng tangerine are inevitably wasted and disposed of annually, resulting in serious environmental pollution and huge economic losses to farmers (Lai et al., 2017; Kahramanolu et al., 2020). Processing berries into wine and other fermented beverages may provide some solutions to circumvent these problems. Brewing wine with Nanfeng tangerines may not only reduce yield loss due to rotting but also increase farmers’ income by promoting value-added products. This strategy can meet the need for high-quality fruit wine and prevent environmental pollution. Fermentation of berries other than grapes, such as cherry, blueberry, blackberry, and raspberry, has recently gained wide interest in response to the growing need of consumers for diversified berry wines (Liu et al., 2018). However, tangerine wine is still not widely produced. Only few studies focused on fermentation optimization (Liao and Wang, 2014; Chen et al., 2015) and Non-*Saccharomyces* isolation (Yu et al., 2019) for high-quality tangerine wine production. Further research is warranted to support the industrial manufacturing of this product.

The microbiota in natural environments is complex and often includes thousands of genera from a diverse range of species (Astudillo-García et al., 2017). In natural environments or under artificially controlled conditions, fermentation microbiota can potentially utilize different raw materials to produce various metabolites (Lu et al., 2016). These metabolites are commonly characterized by the presence of specific microorganisms, orderly microbial succession, and an unusual functional shift (Cagno et al., 2014; Kong et al., 2014). However, the core microbiota that influence the quantity and quality of tangerine wine are poorly understood (Cocolin et al., 2013). Traditionally, species were isolated and identified from food fermentation processes via culture-dependent techniques. However, the knowledge gained by these previous studies about the core structural microbiota in fermented foods remains limited (Wu et al., 2013). With the development of high-throughput sequencing (HTS) techniques, complex microbial communities can now be identified (Sinclair et al., 2015; Wei et al., 2018).

The community structure of the core microbiota in fermented foods and the correlation between microbiota and metabolites during different fermentation processes are important (Papagianni, 2014). Humans have been making wine for thousands of years, and microorganisms play an integral part in this process as they not only drive fermentation but also significantly influence the flavor, aroma, and quality of finished wines (Morrison-Whittle and Goddard, 2018). The diversity and evolution of microorganisms during fermentation of fruit wine, such as grape wine (Portillo and Mas, 2016; Tapsoba et al., 2016) and Palm Wine (Djeni et al., 2020), had attracted more scientists’ attention. Many different species of fungi and bacteria are found naturally associated with fruits and their fermentation (Chanprasartsuk et al., 2010; Nicholas et al., 2014; Liu et al., 2016).

Tangerine wine is a beverage with low alcohol content and variety of flavor components through a series of biochemical reactions. The fermentation mechanism required for tangerine wine therefore involves complex microbiota and metabolites. Moreover, the fermentation microbiotas use tangerine juice as raw materials to form a variety of flavor components through a series of biochemical reactions. However, the functional correlation between the core microbiota and important metabolites remains to be established in tangerine wine. More specifically, microbial succession and the functional shift in the core microbiota tangerine wine have not been clarified to date. Spontaneous fermentation (SF) and inoculated fermentation (IF) are the two main fermentation methods used in producing tangerine wine. Therefore, in this study, we employed several HTS technologies (16S rRNA gene amplicon sequencing, ITS amplicon sequencing, and metatranscriptomics sequencing) to explore the structure and function of core microorganisms in fermentation microbiota. Moreover, we combined ultraperformance liquid chromatography (UPLC) and headspace-gas chromatography-mass spectrometry (HS/GC-MS) to explore the fluctuations in major flavor components. On the basis of this information, we explored the correlations between the core microbiota and important metabolites of Nanfeng tangerine wine.

## Materials and Methods

### Sample Collection

Tangerines (Nanfeng tangerine) were harvested at optimal maturity from Nanfeng County, Jiangxi Province, China, in 2016. The fresh tangerine fruits were washed, squeezed to obtain the juice, with 97 g/L total sugar, 12 ^*o*^Brix soluble solids, and 8.3 g/L citric acid, and no sulfur dioxide was added into the juice. SF was induced spontaneously by the indigenous microbes in tangerine in a 500 m^3^ fermenter, and IF was carried out in a 500 m^3^ fermenter with 5 kg Angel^®^ commercial *Saccharomyces cerevisiae* (for fruit wine, Angel yeast Co., Ltd., China) as the population of cell in the fermentation broth was about 1 × 10^6^ cfu⋅mL^–1^. The loading volume was both approximately 80% of the tank capacity. SF and IF were both conducted at a controlled temperature of 25°C for 10 days. Samples were collected every 2 days for genomic DNA extraction, main products, and flavor component analysis after fermentation. Each sample was analyzed in triplicates.

### Analysis of Chemical and Physical Properties

For the detection of chemical concentration, the samples were centrifuged at 4°C at 8,000 rpm for 5 min. Total sugar content was determined using the concentrated sulfuric acid-phenol method, and ethanol content was measured by gas chromatography (GC). For the detection of organic acids, the samples were centrifuged and filtered through a 0.22 μm MCE filter, and metabolite contents were quantified via UPLC. An Ultimate AQ-C18 column measuring 4.6 mm × 250 mm was used. Refractive index was monitored using a refractive index detector (RID). As the mobile phase, 0.025% trifluoroacetic acid solution and methanol isocratic elution at a ratio of 95:5 were added at a rate of 0.8 mL/min. Column temperature was set to 25°C. Absorbance was monitored at 210 nm by using a Ultraviolet–visible spectroscopy (UV/VIS) detector. For the detection of volatile compounds, the samples were quantified via HS-GC-MS by using an Agilent GC (7,000C) equipped with an Agilent mass selective detector (GC-MSD). The target analytes were separated using a DB-FFAP column (30 m length × 0.25 mm ID × 0.32 μm film thickness; Agilent, CA, United States). The MS source temperature was 230°C, and the mass (MS) quad temperature was 180°C. All mass spectra were acquired in electron impact mode at 70 eV via full scan within the scanning range of 30–500 amu.

### DNA Extraction, PCR Amplification, and MiSeq Sequencing

Genomic DNA was extracted from 33 samples taken from five fermentation stages of SF and IF by using E.Z.N.A. soil DNA Kit (OMEGA, United States). For prokaryotes, the V3 hypervariable region of 16S rRNA genes was amplified using the universal primers 338F (5′-ACTCCTACGGGAGGCAGCAG-3′) and 806R (5′-GACTACHVGGGTWT CTAAT-3′). For eukaryotes, the ITS1 region of fungal rRNA gene was amplified using the universal primers ITS3F (5′-GCATCGATGAAGAACGCAGC-3′) and ITS4R (5′-TCCTCCGCTTATTGATATGC-3′). Primers 338F and ITS1 were added with barcodes. PCR was conducted in triplicate in a 25 μL reaction mixture containing 1 μL of each primer, 2.5 μL of 10 × Pyrobest reaction buffer, 2 μL of dNTPs (2.5 mmol/L), 0.4 μL of Pyrobest DNA Polymerase (Takara), and 15 ng of template DNA. The amplification program consisted of an initial denaturation step at 95°C for 2 min, 35 amplification cycles (each cycle consisted of 95°C for 30 s, 55°C for 1 min, and 72°C for 30 s), and a final incubation of 72°C for 10 min. The amplicons were confirmed by electrophoresis agarose gels (2% agarose in TAE buffer) and purified with AxyPrepDNA gel extraction kit. The amplified products were sent to the Illumina Miseq sequencing platform for sequencing. The raw data for both experiments have been deposited at the China National GeneBank DataBase (CNGBdb) with accession number CNP0001643.

### Sequencing Data Analysis

Raw sequences were sorted with their unique barcodes. Sequences with low quality, read length below 300 bp, and average base quality score of less than 20 were filtered out. Chimera sequences were removed using the Uchime algorithm. The sequences were clustered into operational taxonomic units (OTUs) at 97% identity threshold. The most frequently occurring sequence was extracted as the representative sequence for each OTU and was screened for further annotation using the bacterial SILVA database (Release132, for 16S) (Quast et al., 2012) and the UNITE database (Release 7.2, for ITS) (Kõljalg et al., 2013) with the confidence threshold set to default to ≥0.5. Each sample was rarefied to the same number of reads for both alpha-diversity and beta-diversity (PCoA, UniFrac) analyses.

### Statistical Analysis

Data of volatile compounds were processed using the SPSS version 20.0 statistical package. Principal Component Analysis (PCA) was performed using MATLAB software (version 7.0; Mathworks Inc., Natick, MA, United States) to recognize the wine with different volatile flavors. Heatmap analyses were performed using the OmicShare tools^1^. Calculate the Spearman correlation between microorganisms and metabolites (ρ), find the connection with ρ > 0.5 and *P* < 0.05 as visible object. For co-occurrence between microorganisms analysis, calculate the Spearman coefficient between the microorganisms correlation, and then select | ρ| > 0.5 and *P* < 0.05 as the interaction object. To characterize the contribution of microorganisms to metabolites and co-occurrence between microbial genera and major metabolites, the correlation networks between the selected flavor metabolites and microbial community were visualized via the Cytoscape software (version 3.7.2).

## Results

### Microbial Community Structure and Diversity

A total of 569,834 and 572,200 sequence reads were obtained from the bacteria and fungi samples, respectively. A total of 57 bacterial OTUs were obtained, and 52 fungal OTUs were observed from all samples on the basis of 97% similarity. The rarefaction curves and Simpson diversity indices for bacteria and fungi are shown in Supplementary Figure 1. Although the rarefaction curves were not parallel to the *x*-axis, the Simpson diversity indices reached saturation, suggesting that additional phenotypes could be added with additional sequencing. Nevertheless, the great majority of microbial diversity was captured. The various diversity indices, such as Shannon, Simpson, and Chao1, of bacteria and fungi at different fermentation stages are shown in Table 1. The Shannon diversity index of bacteria in SF initially increased and then decreased, whereas that of fungi decreased during SF. In IF, the Shannon diversity index of bacteria increased and then decreased at the end of the process, whereas that of fungi slightly decreased and then increased. The indices revealed that the microbial community structure during tangerine wine fermentation was continuously adjusted, balanced, and stable.

**TABLE 1:** Sample statistical information about Illumina sequencing results.

Samples	Sequence		OTUs		Shannon		Simpson		Chao1	
	16S	ITS	16S	ITS	16S	ITS	16S	ITS	16S	ITS
Juice	47281 ± 566^c^	49736 ± 7330^b,c^	14 ± 0.47^e^	41 ± 3.56^a^	0.994 ± 0.02^c^	1.19 ± 0.01^a^	0.445 ± 0.03^b^	0.36 ± 0.01^f^	19.92 ± 6.52^c^	42.24 ± 4.22^a^
SF 2	42004 ± 1902^c^	36088 ± 5361^c^	17 ± 1.69^d^	13 ± 5.09^b^	1.353 ± 0.17^b^	1.18 ± 0.22^a^	0.355 ± 0.07^b,c^	0.38 ± 0.08^f^	21.28 ± 2.67^c^	29.00 ± 2.54^b^
SF 4	41981 ± 5838^c^	50759 ± 7327^b,c^	15 ± 1.69^d,e^	10 ± 1.24^b^	0.922 ± 0.32^c^	0.63 ± 0.14^b,c^	0.529 ± 0.19^a,b^	0.66 ± 0.12^c,d^	17.40 ± 2.56^c^	9.83 ± 0.43^c,d^
SF 6	40770 ± 4761^c^	49683 ± 6759^b^	15 ± 1.41^d,e^	6 ± 1.69^c^	0.639 ± 0.14^d^	0.65 ± 0.22^b,c^	0.625 ± 0.14^a^	0.64 ± 0.15^c,d^	18.67 ± 1.69^c^	6.67 ± 2.05^d,e^
SF 8	41943 ± 5771^a^	43021 ± 1932^c^	11 ± 0.81^f^	6 ± 1.69^c^	0.673 ± 0.14^d^	0.75 ± 0.18^b^	0.565 ± 0.11^a,b^	0.58 ± 0.14^c,d^	11.83 ± 0.62^d^	7.67 ± 1.65^d,e^
SF 10	43226 ± 4072^c^	49531 ± 6223^b,c^	15 ± 3.09^d,e^	6 ± 2.16^c^	0.730 ± 0.07^d^	0.53 ± 0.10^c^	0.563 ± 0.04^a^	0.68 ± 0.12^c,d^	16.73 ± 2.48^c^	5.67 ± 0.88^e^
IF 2	53235 ± 7663^b,c^	61515 ± 8633^a,b^	23 ± 3.09^c^	9 ± 0.47^b^	1.069 ± 0.14^c^	0.32 ± 0.12^d^	0.451 ± 0.05^b^	0.86 ± 0.06^b^	29.90 ± 8.00^b,c^	11.5 ± 0.70^c^
IF 4	55575 ± 5081^b,c^	67240 ± 9229^a^	35 ± 2.86^a^	5 ± 0.81^c,e^	1.466 ± 0.08^b^	0.05 ± 0.01^e^	0.324 ± 0.04^c^	0.99 ± 0.01^a^	42.67 ± 2.49^a^	6.33 ± 2.63^d,e^
IF 6	64787 ± 4001^b^	59121 ± 10456^a^	27 ± 0.81^b^	5 ± 0.94^c,e^	1.483 ± 0.03^b^	0.69 ± 0.01^b^	0.358 ± 0.01^c^	0.51 ± 0.01^e^	30.98 ± 4.49^a,b^	5.67 ± 1.24^d,e^
IF 8	63945 ± 4153^b^	53943 ± 3557^b^	25 ± 5.43^b,c^	6 ± 0.81^c^	1.500 ± 0.10^a^	0.65 ± 0.02^b^	0.305 ± 0.04^c^	0.56 ± 0.01^d^	29.33 ± 5.39^b^	6.33 ± 0.94^d,e^
IF 10	75087 ± 4258^a^	51563 ± 15400^b^	30 ± 1.24^a,b^	4 ± 0.94^e^	1.042 ± 0.14^c^	0.45 ± 0.07^c^	0.503 ± 0.06^a,b^	0.72 ± 0.06^c^	38.83 ± 10.05^a,b^	4.33 ± 0.94^e^

*SF, spontaneous fermentation; IF inoculated fermentation. The numbers after SF and IF represent the fermentation days. The means and the SD (n = 3) followed by different superscript lowercase letter within the same column denote a significant difference at P < 0.05 of microbial diversity among different samples.*

### Microbial Community Dynamics During Wine Fermentation

**FIGURE 1:**
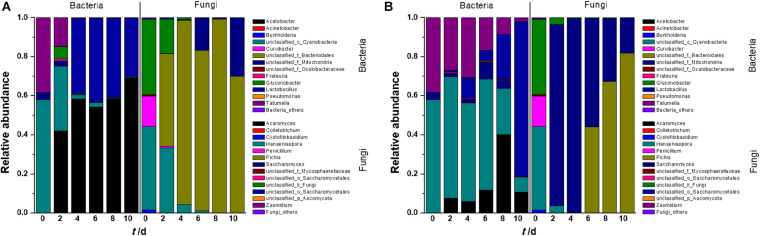
Relative abundance plots of microbial community composition during the entire fermentation period (**A:** SF; **B:** IF). Each value was the mean of triplicate samples.

The microorganisms in fermentation broth were found to belong to five bacterial phyla and two identified fungal phyla (Supplementary Figure 2). At the genus level, 13 bacterial genera were identified in tangerine wine fermentation (Figure 1A). In SF, *Cyanobacteria*, *Tatumella*, *Acetobacter*, and *Gluconobacter* were abundant in the early stage of fermentation, although their abundance gradually decreased as fermentation progressed and *Acetobacter* became predominant during the middle up to the last stages of fermentation. *Lactobacillus* was detected on the 4th day of fermentation and became one of the most dominant genera in the latter stage of fermentation. In IF, *Cyanobacteria* and *Tatumella* were consistently detected with relatively higher abundance throughout the entire IF than that in SF. Interestingly, the majority of OTUs in the last stage of IF belonged to *Lactobacillus*, whereas *Acetobacter* dominated in SF. This result arose likely because of certain wine characteristics, such as the contents of sugar, ethanol, and organic acids. Analyses of both clusters and significant differences revealed a sharp distinction between SF and IF in terms of bacterial communities (Supplementary Figures 3A, 4A). Partial least squares discriminant analysis (PLS-DA) also showed significant differences among the original juice, SF, and IF (Supplementary Figure 5A).

Compared with bacterial diversity, only eight fungal genera were found in tangerine juice (Figure 1B). In SF, *Hanseniaspora* was the dominant genus in the early stage of fermentation, but its proportion sharply decreased from 43 to 5% during the subsequent stages. *Pichia* existed almost throughout the entire fermentation process and became the dominant fungus in the broth. *Saccharomyces*, the inoculant of commercial yeast, dominated the fungal community in the early and middle stages of IF, whereas *Hanseniaspora*, *Pichia*, and *Penicillium* were inhibited during these stages. However, *Saccharomyces* rapidly declined on day six when *Pichia* greatly increased and became the dominant genus in the subsequent IF stages. Cluster analysis revealed an overlapping but clear and remarkable difference in fungal communities between SF and IF (Supplementary Figure 3B). Analysis of significant differences showed that SF and IF were obviously different in terms of the fungal communities of *Saccharomyces* and *Hanseniaspora* (Supplementary Figure 4B). PLS-DA analysis also demonstrated sharp distinctions in fungal microbiota in juice, SF, and IF (Supplementary Figure 5B). The diversity of fungal communities in juice was greater than that in fermentation broth, indicating that the fungi were sensitive to alcohol fermentation.

### General Features of Nanfeng Tangerine Wine Fermentation

The reducing sugar, alcohol content, pH, and organic acids in the fermentation of Nanfeng tangerine wine were investigated to determine its fermentation characteristics (Table 2). As a sweet fruit variety, the sugar content of tangerine juice can reach up to 125.40 g/L. With the juice as the medium, sugar consumption rate was smaller in SF than that in IF, and the concentration of reducing sugars in the broth were 26.94 and 1.39 g/L, respectively, at the end of fermentation. Consequently, ethanol production in IF (4.61%) was higher than that in SF (2.2%), suggesting that the microbial community in IF which dominated by *S. cerevisiae*, customarily used for alcoholic wine fermentation, has excellent performance on sugar utilization and alcohol production.

**TABLE 2:** Chemical and physical properties of samples at different fermentation stages.

Samples	Sugar (g/L)	Ethanol (%)	Lactic acid (g/L)	Acetic acid (g/L)	Citric acid (g/L)	Succinic acid (g/L)
Juice	125.40 ± 5.36^a^	0.02 ± 0.01^d^	0.12 ± 0.02^f^	1.25 ± 0.25^e^	9.95 ± 1.86^c^	0.99 ± 0.12^c^
SF 2	75.00 ± 4.86^b^	0.34 ± 0.06^c^	0.58 ± 0.05^e^	1.43 ± 0.22^e^	15.26 ± 1.12^a,b^	0.38 ± 0.05^d^
SF 4	52.34 ± 5.24^c^	0.35 ± 0.04^c^	1.41 ± 0.54^b^	2.42 ± 0.15^c^	12.01 ± 1.01^b,c^	0.98 ± 0.09^c^
SF 6	45.92 ± 2.59^c^	1.72 ± 0.27^b^	1.86 ± 0.59^b^	2.20 ± 0.23^c^	8.94 ± 0.20^c^	2.76 ± 0.40^a^
SF 8	39.32 ± 2.27^d^	2.15 ± 0.22^b^	3.93 ± 0.21^a^	1.79 ± 0.07^d^	2.26 ± 0.98^a^	1.77 ± 0.31^b^
SF 10	26.94 ± 3.84^e^	2.26 ± 0.28^b^	3.67 ± 0.23^a^	1.51 ± 0.24^d,e^	0.06 ± 0.03^d^	1.65 ± 0.25^b^
IF 2	3.33 ± 0.42^f^	4.72 ± 0.54^a^	0.94 ± 0.07^c,d^	5.51 ± 0.31^b^	15.67 ± 0.76^a^	0.73 ± 0.12^c^
IF 4	1.27 ± 0.27^g,h^	4.81 ± 0.66^a^	1.02 ± 0.11^c^	5.66 ± 0.18^b^	15.66 ± 0.73^a^	1.32 ± 0.31^b,c^
IF 6	1.61 ± 0.14^g^	4.34 ± 0.64^a^	0.94 ± 0.09^c,d^	8.12 ± 0.18^a^	13.36 ± 0.45^b^	1.39 ± 0.06^b^
IF 8	0.19 ± 0.06^i^	4.94 ± 0.54^a^	0.81 ± 0.09^d^	8.92 ± 0.66^a^	13.44 ± 0.69^b^	1.67 ± 0.04^b^
IF 10	1.39 ± 0.08^h^	4.61 ± 0.86^a^	0.80 ± 0.07^d^	8.86 ± 0.61^a^	12.37 ± 0.13^b,c^	1.75 ± 0.30^b^

*SF, spontaneous fermentation; IF inoculated fermentation. The numbers after SF and IF represent the fermentation days. The means and the SD (n = 3) followed by different superscript lowercase letter within the same column denote a significant difference at P < 0.05 of chemical and physical properties among different samples.*

The main organic acids detected in tangerine juice were lactic acid, acetic acid, citric acid, and succinic acid, with a concentration of 0.12, 1.25, 9.95, and 0.99 g/L, respectively (Table 2). In SF, lactic acid concentration gradually increased during fermentation and was higher than that in IF. By contrast, acetic acid production in SF was considerably lower than that in IF during fermentation. The citric acid in both processes accumulated in the early stage of fermentation and then was gradually consumed in the middle and late stages. In particular, the citric acid in SF was almost depleted by the end of fermentation. Succinic acid was continuously consumed and synthesized during fermentation. In IF broth, lactic acid concentration slightly increased in the early stage of fermentation (0–4 days) and then slightly decreased in subsequent stages, possibly because of the esterification of some of the lactic acid during fermentation. Citric acid and succinic acid were maintained at relatively stable concentrations in IF because they are normal fermentation products of alcohol fermentation. In general, IF wine had higher organic acid content than SF probably because of the presence of commercial yeast in IF, which has a high rate of glucose metabolism and leads to accumulation of organic acids.

### Flavor Evolution in Fermentation

**FIGURE 2:**
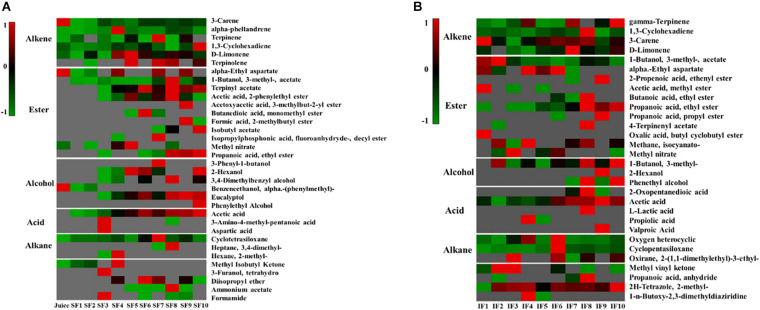
Heatmap of change in metabolic profiling during the fermentation process (**A:** SF; **B:** IF). Clustering analysis was performed using Pearson correlation and Euclidean distance based on the relative content of metabolites during the fermentation process. Data were processed by z-score transformation.

Tangerine wine fermentation can produce large amounts of volatile aromatic compounds, such as alkanes, alkenes, esters, alcohols, and acids. The main aromatic components of Nanfeng tangerine juice were alkenes and alkanes, including 3-decene, α-phellandrene, terpinene, 1,3-cyclohexadiene, d-limonene, cyclotetrasiloxane, and small amounts of esters and alcohols, such as α-ethyl aspartate, methyl nitrate, and α-methyl-benzeneethanol (Figure 2).

**FIGURE 3:**
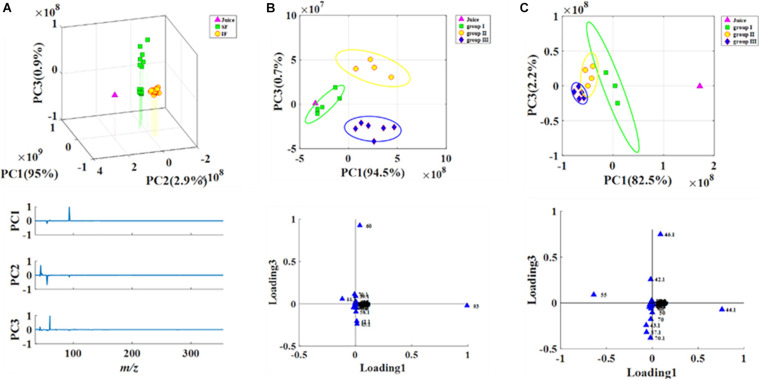
PCA analysis of volatile compounds in fermentations (**A:** Juice, SF, and IF; **B:** SF; **C:** IF).

Headspace-gas chromatography-mass spectrometry was performed to evaluate the metabolic profiles of the volatile compounds of SF and IF. PCA illustrated that the samples collected from SF and IF formed a group in the negative quadrants of PC1, PC2, and PC3. Three PCs (PC1 eigenvalue, 95%; PC2 eigenvalue, 2.9%; PC3 eigenvalue, 0.9%) representing about 98.8% of the total variance (Figure 3A) were selected for modeling. SF samples were more scattered than IF samples, indicating that the volatile compounds of SF were more variable than those of IF. The primary characteristic substances in both SF and IF were acetic acid, isoamyl acetate, and terpinyl acetate. In SF, these samples could be divided into three groups (group I: SF 1–3 days; group II: SF 4–7 days; group III: SF 8–10 days) according to their compounds’ loadings on the axes (Figure 3B). Each group was completely separated. In the early stage of fermentation (group I), small amounts of acids and esters, such as acetic acid, 3-amino-4-methyl-pentanoic acid, aspartic acid, 1-butanol 3-methyl-acetate, and terpinyl acetate, were formed. As the fermentation progressed, several alcohols, including 2-hexanol, eucalyptol, and 3,4-dimethylbenzyl alcohol, were synthesized. The content of acetic acid and terpinyl acetate gradually increased, and certain esters, such as acetic acid 2-phenylethyl ester, butanedioic acid monomethyl ester, and propanoic acid ethyl ester, were formed. The contents of aromatic compounds 1-butanol 3-methyl-acetate, acetic acid, eucalyptol, and terpinyl acetate gradually increased in the late stage of SF, whereas certain esters, such as formic 2-methylbutyl ester, isobutyl acetate, and propanoic acid ethyl ester, were produced. According to the corresponding loading plots, the primary substances that substantially influenced the changes in groups were terpinyl acetate, acetic acid, 1-butanol, and 3-methyl-acetate (Figure 3B). Results showed that the content of acetic acid in SF wine was comparatively high and thus negatively affected the quality of wine.

IF was also divided into three groups (group I: IF 1–2 days; group II: IF 3–6 days; group III: IF 7–10 days) (Figure 3C). In the early stage of IF, various alcohols and esters, such as 1-butanol 3-methyl-acetate, acetic acid methyl ester, propanoic acid ethyl ester, isocyanato-methane, and ethyl nitrate, were synthesized. In the middle stage, the content of acetic acid increased, whereas that of acetic acid and methyl ester decreased. Moreover, the traditional aromatic components of IF in the middle and late stages of fermentation were quite similar according to principal component plots. Small amounts of phenethyl alcohol formed in the late stage of fermentation.

### Correlation Between Microbial Communities and Metabolites

**FIGURE 4:**
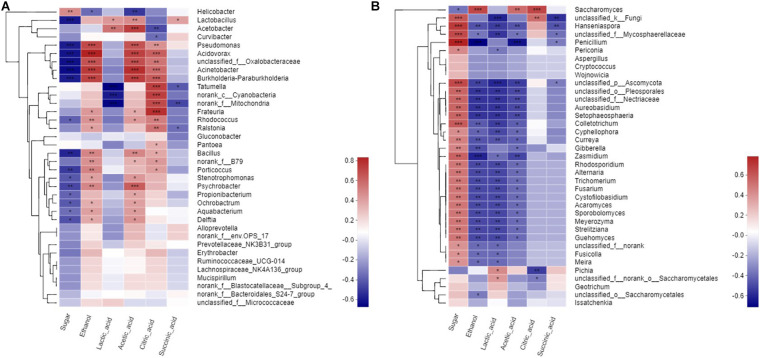
Spearman corelation heatmap of metabolites to microbial. **(A)** a prokaryotic community, **(B)** eukaryotic community at genus level.

Mantel test was performed to assess the significance of Spearman’s correlation on the basis of bacterial and fungal communities and metabolites and thus elucidate the relationships between these elements. The bacteria in wine fermentation were significantly positively correlated with ethanol, acetic acid, and citric acid but negatively correlated with sugar content (Figure 4A). *Acetobacter* was negatively associated with citric acid but positively correlated with lactic acid. *Lactobacillus* was positively correlated to acetic acid and lactic acid. Except for *Saccharomyces* and *Pichia*, the fungi had a significantly positive correlation with sugar. Furthermore, most of the fungi were negatively correlated with ethanol, lactic acid, and acetic acid (Figure 4B). However, *Saccharomyces* was significantly positively correlated with ethanol. *Hanseniaspora* was significantly negatively correlated with ethanol, lactic acid, acetic acid, and succinic acid but significantly positively correlated with sugar. *Penicillium* was significantly negatively correlated with ethanol and acetic acid but positively correlated with sugar; however, this correlation gradually diminished as fermentation progressed. *Saccharomyces* was significantly positively correlated with ethanol, acetic acid, and citric acid. *Pichia* was significantly positively correlated with lactic acid but significantly negatively correlated with citric acid.

**FIGURE 5:**
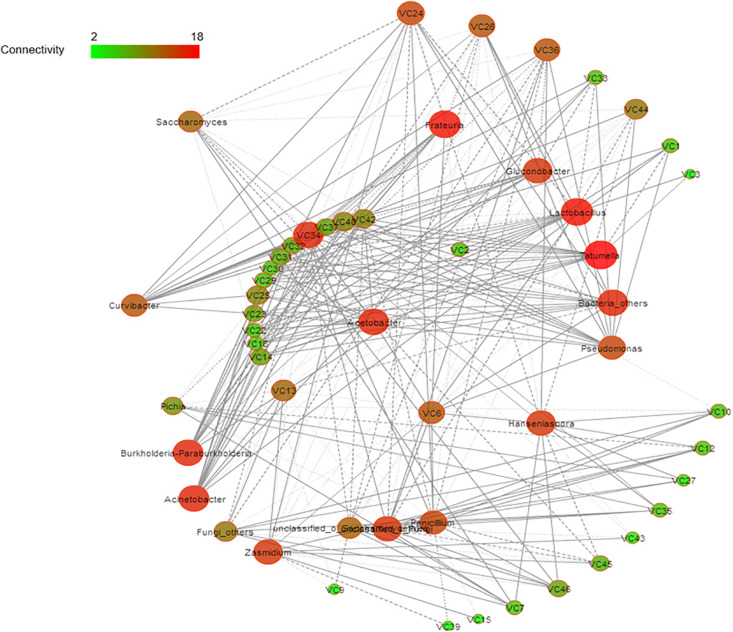
Correlation network of co-occurring microbial genera in fermented grains. Statistically significant (*P* < 0.05) Spearman correlation coefficient (| ρ| > 0.5) indicates the robust correlations. The size of nodes indicates the degree of connections. The same color of nodes indicates the same order. Edge thickness represents the proportional to the value of Spearman’s correlation.

Spearman correlation and diversity analyses were combined to explore the positive and negative correlations between the core microbiota and volatile compounds (Figure 5); | ρ| > 0.5 was selected as the interaction object. In general, a significant and robust relationship (edge) was established between 18 genera (nodes) and 34 metabolites (*P* < 0.05). *Lactobacillus*, *Acetobacter*, *Hanseniaspora*, and *Saccharomyces* were the largest contributors to the production of the volatile flavor of Nanfeng tangerine wine. At the genus level, the bacteria *Lactobacillus*, *Acetobacter*, and *Tatumella* and the fungi *Hanseniaspora*, *Saccharomyces*, and *Pichia* were identified as the core functional microbiota for the production of the volatile flavor of Nanfeng tangerine wine.

Spearman analysis identified *Lactobacillus* as the core microbe that enhances the production of volatile flavor. *Lactobacillus* was strongly positively associated with esters, including terpinyl acetate, methyl nitrate, and acetic acid 2-phenylethyl ester. *Acetobacter* was significantly positively correlated with isobutyl acetate, 1-butanol 3-methyl-acetate, propanoic acid ethyl ester, as well as with acids and alcohols, such as acetic acid, eucalyptol, 3,4-dimethylbenzyl alcohol, and 2-hexanol. *Hanseniaspora* was correlated with esters, such as methyl nitrate, butanoic acid ethyl ester, terpinenyl acetate, and propanoic acid ethyl ester. Furthermore, *Saccharomyces* was positively correlated with terpinyl acetate, 1-butanol, 3- methyl-, and acetic acid. However, *Pichia* had partially contributed to the volatile flavor of Nanfeng tangerine wine and was mainly related to butanoic acid ethyl ester, acetic acid, and terpinyl acetate.

## Discussion

Fruits wines are characterized by peculiar aromatic notes, antioxidant potential, phenolic composition, alcohol content, and other parameters. Using tangerine juice as a raw material to brew a unique flavor of fruit wine could help to reduce postharvest and production losses of tangerine. Some efforts have been made to optimize the fermentation process and use Non-*Saccharomyces* to improve the quality of tangerine wine (Liao and Wang, 2014; Chen et al., 2015; Yu et al., 2019). As the basic processes of fruit wines are similar to grape juice production and fermentation, both endogenous and exogenous microorganisms are involved in the process of fruit wine fermentation. Understanding the diversity and evolution of microorganisms during fruit wine fermentation is essential for controlling its production. Recent studies focused on the microbial composition and diversity during the wine fermentation based on culture-dependent methods (Ghosh et al., 2015; Liu R. et al., 2015) or culture-independent methods (Portillo and Mas, 2016; Tapsoba et al., 2016; Wei et al., 2018). These diverse microbial populations and their potential interplay in the different stages of alcoholic fermentation constitute a complex biological process that contributes largely to the final chemical composition (Gammacurta et al., 2017) of wine and, therefore, to its sensory properties (Hu et al., 2020). SF and IF are the main fermentation methods employed in producing wine. Analysis obtained from spontaneous and IFs has shown significant differences in the chemical composition and sensory properties of wine (Blanco et al., 2008). Exploring the microbial diversity and dynamics in different fermentation methods of wine is essential both to understand the role of core functional microbiota in the process, and to select autochthonous starter strains that are potentially able to contribute wines with unique regional characteristics, avoiding the risks associated with SFs (Knight et al., 2015; Capozzi et al., 2020). In this study, we systematically investigated the dynamics of microbial communities and metabolites during SF and IF. To enhance our understanding of the microbial dynamics during tangerine fermentation, we employed 16S rRNA gene and ITS amplicon sequencing (Benjamin et al., 2014) and determined the changes in bacterial and fungal communities during fermentation. The ITS sequence is more suitable for the analysis of fungal communities because its highly variable regions are more taxonomically informative than 18S rDNA variable regions.

Diversity indices indicated that the diversity of bacterial communities in IF was more abundant than that in SF. In SF, the diversity of bacterial communities initially increased and then decreased presumably because the high content of nutrients in the juice was suitable for bacterial growth. By contrast, the environmental conditions during the late fermentation stage and yeast growth had a certain inhibitory effect on bacterial growth. In IF, bacterial diversity remained stable, suggesting that the bacteria were able to tolerate the highly acidic and highly ethanolic fermentation environment. The diversity of bacteria in IF was more abundant than that in SF. In terms of bacterial communities, 13 genera were identified during tangerine wine fermentation. *Acetobacter*, *Lactobacillus Gluconobacter*, and *Cyanobacteria* are frequently detected in wine (Liu S. et al., 2015; Piao et al., 2015; Chen et al., 2017). The high abundance of *Lactobacillus* in IF might be related to the fact that, in wine, *S. cerevisiae* inoculation can modulate the bacterial consortium, improving the concentration of protechnological bacteria (Berbegal et al., 2019). *Acetobacter* is moderately resistant to alcohol; thus, this bacterium was detected in the late stages of fermentation. Although *Acetobacter* has been identified as a wine spoilage bacterium and is rarely observed in wine, several studies also detected *Acetobacter* during wine fermentation, suggesting that the population of acetic acid bacteria might have often been underestimated via culture-dependent methods because of the lack of appropriate cultivation techniques (Millet and Lonvaud-Funel, 2010). *Lactobacillus*, which can convert malic acid to lactic acid and enhance the taste and sensory quality of wine (Gammacurta et al., 2018), was the dominant bacterium in SF wine. Lactic acid has a buffering effect on blending wine taste, and it can be synthesized as a series of lactate esters with alcohol to enrich the esters in wine (Liang et al., 2015). The observed increase in *Lactobacillus* abundance at the late stage of IF could be expected to generate the appropriate conditions for subsequent malolactic fermentation (Portillo and Mas, 2016). *Gluconobacter*, as a wine spoilage bacterium, is often found in grapes and fruit surfaces. *Gluconobacter* preferentially utilizes sugar as a carbon source, whereas *Acetobacter* prioritizes the use of alcohol; hence, the latter is more resistant to alcohol than the former, an observation reported in previous studies (Piao et al., 2015). This fact may explain why the abundance of *Gluconobacter* detected in SF wine was higher than that in IF wine. A comparison of the dynamics of bacterial communities in SF and IF that the abundance of *Cyanobacteria* and *Tatumella* was significantly higher in the middle stage of IF than that at the same stage in SF probably because these bacterial genera were inhibited by *Acetobacter*.

The diversity of fungal communities in both SF and IF decreased with the progress of fermentation suggesting that the fungi involved in brewing might not be adaptable to the highly acidic and highly ethanolic fermentation environment. Harsh conditions reportedly inhibit fungal communities until they become adapted to, or even tolerant of, the harsh fermentation environment (Wang and Liu, 2013). The most abundant fungal genera during SF were *Hanseniaspora*, *Pichia*, and *Saccharomyces* with a clear succession during SF, whereas those during IF were *Saccharomyces*, *Hanseniaspora*, and *Pichia*. These fungal genera have been frequently isolated in wine fermentation (Liu R. et al., 2015). The abundance of *Hanseniaspora* gradually decreased during fermentation because it is sensitive to ethanol, as was observed in previous studies (Moreira et al., 2011; Raymond et al., 2017). The major difference between SF and IF of Nanfeng tangerine wine employed in this study was the addition of commercial yeast; thus, *Saccharomyces* dominated at the pre-fermentation stage of IF. Interestingly, *Pichia* began to appear on the 6th day of IF and gradually dominated in the late fermentation stage. This result was somewhat unexpected because *Pichia* is moderately resistant to alcohol and usually detected in the middle stage of fermentation (Portillo and Mas, 2016). Some *Pichia* species in Nanfeng tangerine wine probably had a better ethanol tolerance than *Saccharomyces* or they were more adaptable to the environment of the late fermentation period. This finding was similar to that of several studies that reported that some strains of *P. pastoris* have better ethanol tolerance than *Saccharomyces* (Benito et al., 2011; Portillo and Mas, 2016). *Saccharomyces* became predominant at the middle and late stages of fermentation because of its efficient fermentation catabolism and ethanol tolerance and highly competitive ability (Wang and Liu, 2013; Huang et al., 2018). In this study, *Saccharomyces* was detected in the late stage of SF unlike that in previous studies. Previous studies argued that *Saccharomyces* rarely appears on the surface of berries and hardly exists in citrus wine SF (Barata et al., 2012; Liu R. et al., 2015). As important generators of various secondary metabolites, the other fungal genera were dominant in the pre-fermentation stage of Nanfeng tangerine wine, implying that these genera can be used to produce large amounts of flavor substances and thus improve wine quality.

Wines produced via IF and SF had a final ethanol concentration of 2.2 and 4.61%, respectively. The content of residual sugar in IF (1.39 g/L) was considerably lower than that in SF (26.94 g/L). These results suggested that IF can generate Nanfeng tangerine wines with a higher alcohol level and a lower residual sugar level than SF. The total acid content was higher in IF than in SF; the contents of acetic acid and citric acid were substantially higher in IF than those in SF, whereas that of lactic acid was more abundant in SF than that in IF likely because of the complex microbial activities involved in fermentation (Cai et al., 2018). In this work, Mantel test was performed to assess the significance of Spearman’s correlation on the basis of bacterial and fungal communities and metabolites. Results showed that the bacteria were significantly positively correlated with citric acid, ethanol, and acetic acid but negatively correlated with sugar content. In addition, acetic acid and lactic acid production was correlated with *Lactobacillus*, and the increase in acidity was apparently correlated with the abundance of *Acetobacter*, a result that was consistent with that of previous studies (Papalexandratou et al., 2013; Tapsoba et al., 2016; Gammacurta et al., 2018). Correlation analysis revealed that *Acetobacter* was strongly correlated with acetic acid possibly because the pathway of acetic acid production was enhanced by *Acetobacter* in Nanfeng tangerine wine fermentation. Except for *Saccharomyces*, almost all of the fungi were negatively correlated with ethanol. This observation can explain the fact that yeast succession in wine concerning other fungal genera is followed by *S. cerevisiae* after the middle stage of fermentation (Zhang et al., 2017; Cai et al., 2018). *Saccharomyces* was significantly positively correlated with ethanol, acetic acid, and citric acid, a finding that explained why the contents of ethanol, acetic acid, and citric acid were substantially higher in IF than in SF. *Pichia* was the main contributor to lactic acid and significantly negatively correlated with citric acid. Previous studies reported that some *Pichia* species, such as *P. kudriavzevii*, can degrade L-malic acid, increase pH, and produce low levels of ethanol and acceptable amounts of acetic acid (Mónaco et al., 2014).

In the present study, 34 and 36 volatile compounds were detected in SF and IF, respectively. PCA showed significant differences between SF and IF. The wine produced via SF had distinct aromatic profiles unlike that produced via IF. SF wine was characterized by abundant esters and high amounts of volatile acids, whereas IF wine contained high amounts of alcohols, such as isoamyl alcohol. The content of acetic acid in SF wine was relatively higher than that in IF wine that had adverse effect on quality. In addition, SF wine contained high amounts of esters, including terpinyl acetate, 1-butanol 3-methyl-acetate, acetic acid 2-phenylethyl ester, butanedioic acid monomethyl ester, propanoic acid ethyl ester, formic acid 2-methylbutyl ester, and isobutyl acetate. These esters impart a unique fruit aroma to wines, and they play an important role in the formation of the overall aromatic profile of tangerine wine. Small amounts of isoamyl alcohol, which is an important flavor in wine, can impart an elegant aroma to wines. However, amounts of this alcohol beyond the sensory threshold adversely affect the flavor of wines and may even cause poisoning. Deficiency in volatile aroma is one of the limiting factors of wine production. The volatile aromatic profiles of fruit wines can be improved by inoculating the fermentation with Non-*Saccharomyces* yeast.

The relationship between microbiota succession and volatile flavor dynamics during SF and IF of Nanfeng tangerine wine remains unclear. The core functional microbiota responsible for the production of volatile flavor can be explored by considering both dominance and functionality. In this study, a significant and robust relationship (edge) was established between 18 genera (nodes) and 34 metabolites (*P* < 0.05). *Lactobacillus*, *Acetobacter*, *Hanseniaspora*, and *Saccharomyces* were the largest contributors to the production of the volatile flavor of Nanfeng tangerine wine. *Lactobacillus* became the predominant bacterium in wine and it was the major producer of lactic acid and acetic acid under strict anaerobic conditions during wine fermentation (Song et al., 2017). Strict anaerobic conditions ensure the stability of wines and improve their aromas and flavors by enhancing ester production (Wang et al., 2014; Song et al., 2017; Gammacurta et al., 2018; Xu et al., 2018). *Hanseniaspora* was the main contributor to some esters (Tufariello et al., 2021), a finding that explained why the esters in IF wine were poor; the wild yeasts in Nanfeng tangerine likely had difficulty competing against the commercial yeast. Aside from *Saccharomyces*, the other indigenous fungal genera could adapt well to the local fermentation conditions and substantially contribute to the characters of specific regional aroma. Nonetheless, the actual contribution of yeasts other than *Saccharomyces* to the final volatile flavor of Nanfeng tangerine wine requires further study. Moreover, the mixed culture of *Saccharomyces* and other fungal genera is a valuable tool for modulating the volatile profiles and improving the aromatic complexity of wine (Liu et al., 2016). Therefore, further research is warranted to highlight the beneficial enological traits of non-*Saccharomyces* yeasts. This end may be achieved via sequential co-fermentation with *S. cerevisiae* or by changing the inoculation time and ratio of non-*Saccharomyces* yeasts.

## Conclusion

Spontaneous fermentation could improve the aromatic profiles of Nanfeng tangerine wine. Non-*Saccharomyces* yeasts and *Lactobacillus* played an important role in determining the aromatic and flavor profile of wine produced via SF by producing abundant esters and high amounts of volatile acids. The results of the present study can be used to develop effective strategies for selecting beneficial microorganism to improve the quality of Nanfeng tangerine wine.

## Data Availability Statement

The datasets presented in this study can be found in online repositories. The names of the repository/repositories and accession number(s) can be found below: China National GeneBank DataBase (CNGBdb) (https://db.cngb.org/), accession number CNP0001643.

## Author Contributions

XQ: conceptualization, methodology, validation, investigation, and writing – original draft. LY: investigation, methodology, validation, and writing – review and editing. WW: data curation and software. RY: conceptualization, writing – review and editing, visualization, project administration, and funding acquisition. ZZ: investigation and methodology. HY: investigation. DZ: conceptualization, methodology, project administration, and funding acquisition. BZ: resources. All authors contributed to the article and approved the submitted version.

## Conflict of Interest

The authors declare that the research was conducted in the absence of any commercial or financial relationships that could be construed as a potential conflict of interest.

## Footnotes

1 http://www.omicshare.com/tools
