# Supplementary material for: Comparative Evaluation of Microbiota Dynamics and Metabolites Correlation Between Spontaneous and Inoculated Fermentations of Nanfeng Tangerine Wine
Source: Front Microbiol. 2021 May 11;12:649978. doi: 10.3389/fmicb.2021.649978 (PMC8144288; doi:10.3389/fmicb.2021.649978)
Supplement: Supplementary file 1 [file Data_Sheet_1.docx]

# *Supplementary Material*

**This file includes:**

1. Supplementary Figures (Figure S1- Figure S10)

2. Supplementary Tables (Table S1- Table S2)


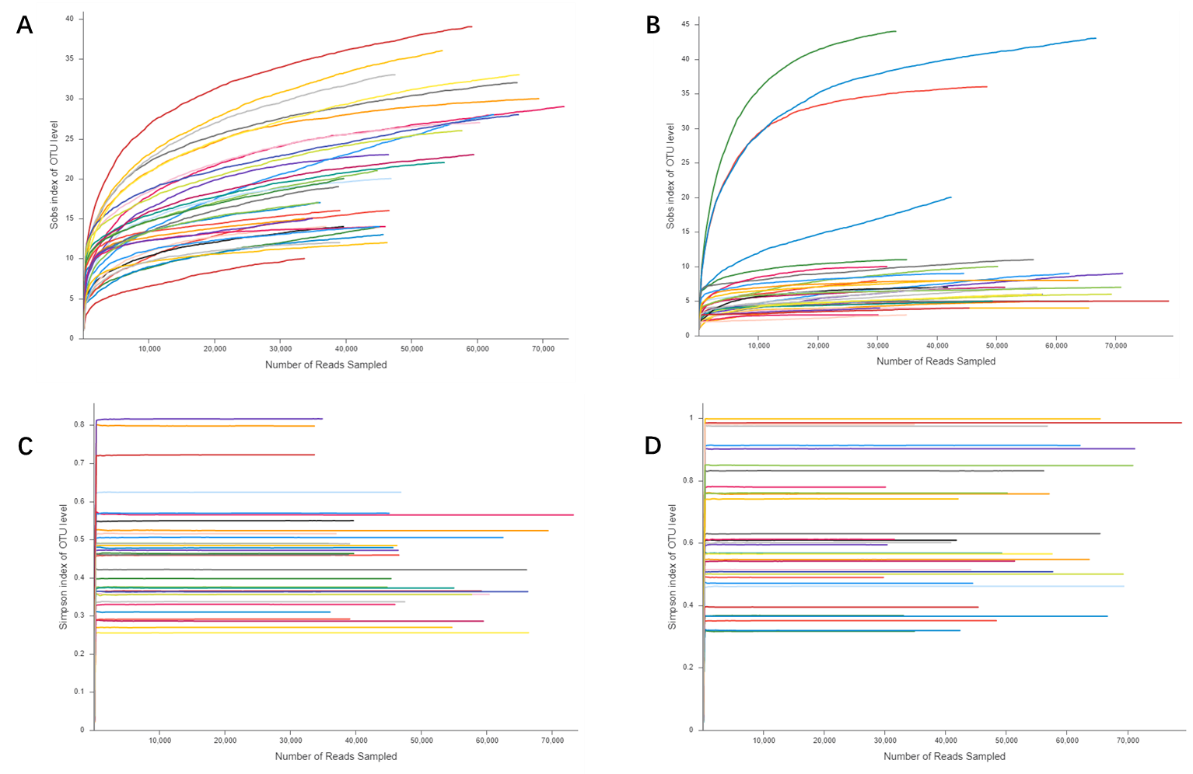


**Figure S1.** Rarefaction cure (A: bacteria; B: fungi) and Simpson diversity (C: bacteria; D: fungi) in tangerine fermentation wine.


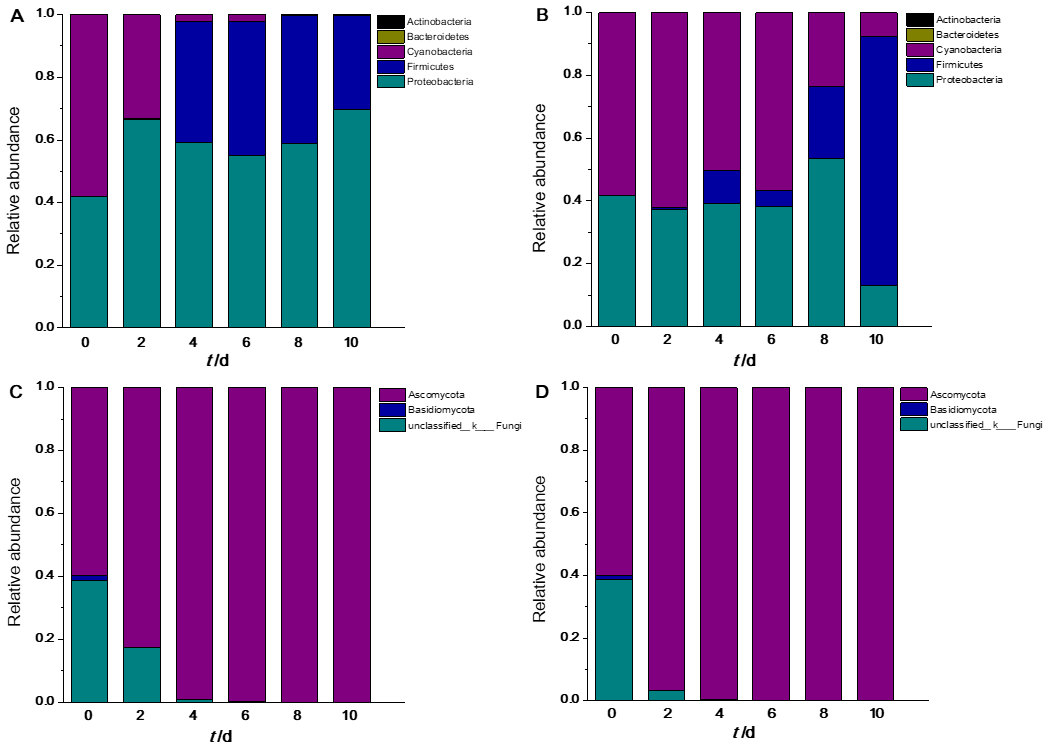


**Figure S2.** Relative abundance of bacteria (A: SF; B: IF) and fungi (C: SF; D: IF) at phyla levels.

**
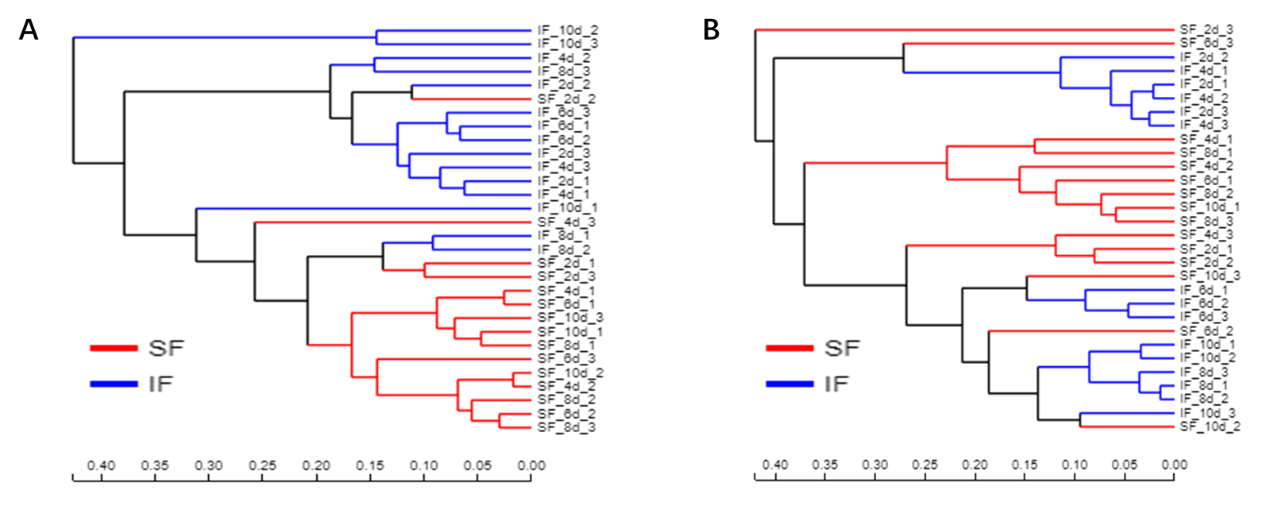
**

**Figure S3.** Cluster analysis of the microbiota (A: bacteria; B: fungi) in SF and IF based on beta diversity distances.


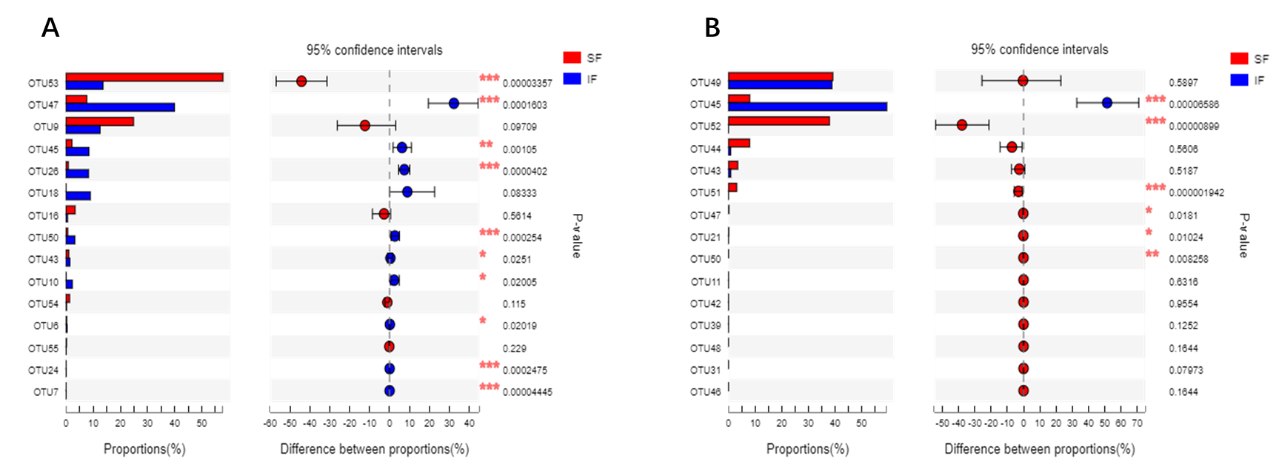


**Figure S4.** Significant analysis of the microbiota (A: bacteria; B: fungi) in SF and IF.


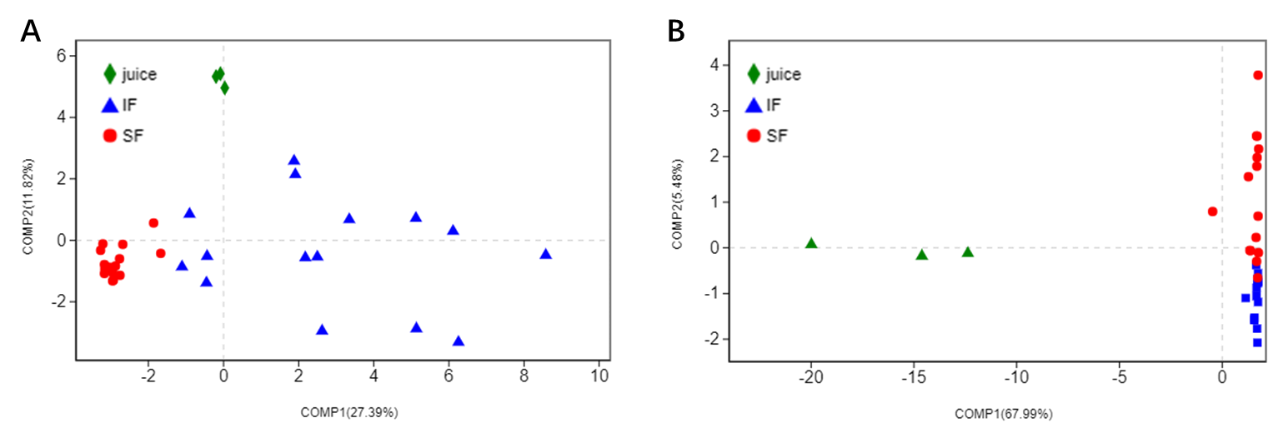


**Figure S5.** Partial least squares discriminant analysis of the microbiota (A: bacteria; B: fungi) in SF and IF.

**2. Supplementary Table**

**Table S1.** Abbreviation of volatile compounds in the correlation network (for Figure 5)

| **abb.** | **Compunds** | **abb.** | **Compunds** |
| --- | --- | --- | --- |
| VC1 | Terpinyl acetate | VC24 | D-Limonene |
| VC2 | Terpinolene | VC25 | Diisopropyl ether |
| VC3 | Terpinene | VC26 | Cyclotetrasiloxane |
| VC4 | Propiolic acid | VC27 | Butanoic acid, ethyl ester |
| VC5 | Propanoic acid, propyl ester | VC28 | Butanedioic acid, monomethyl ester |
| VC6 | Propanoic acid, ethyl ester | VC29 | Benzeneethanol, alpha.-(phenylmethyl)- |
| VC7 | Propanoic acid, anhydride | VC30 | Ammonium acetate |
| VC8 | Phenylethyl Alcohol | VC31 | alpha-phellandrene |
| VC9 | Oxygen heterocyclic | VC32 | alpha-Ethyl aspartate |
| VC10 | Oxirane, 2-(1,1-dimethylethyl)-3-ethyl-, | VC33 | Acetic acid, 2-phenylethyl ester |
| VC11 | Oxalic acid, butyl cyclobutyl ester | VC34 | Acetic acid |
| VC12 | Methyl vinyl ketone | VC35 | 4-Terpinenyl acetate |
| VC13 | Methyl nitrate | VC36 | 3-Carene |
| VC14 | Methyl Isobutyl Ketone | VC37 | 3,4-Dimethylbenzyl alcohol |
| VC15 | Methane, isocyanato- | VC38 | 2-Oxopentanedioic acid |
| VC16 | L-Lactic acid | VC39 | 2H-Tetrazole, 2-methyl- |
| VC17 | Isopropylphosphonic acid, fluoroanhydryde-, decyl ester | VC40 | 2-Hexanol |
| VC18 | Isobutyl acetate | VC41 | 1-n-Butoxy-2,3-dimethyldiaziridine |
| VC19 | Hexane, 2-methyl- | VC42 | 1-Butanol, 3-methyl-, acetate |
| VC20 | Heptane, 3,4-dimethyl- | VC43 | 1-Butanol, 3-methyl- |
| VC21 | Formic acid, 2-methylbutyl ester | VC44 | 1,3-Cyclohexadiene |
| VC22 | Formamide | VC45 | gamma-Terpinene |
| VC23 | Eucalyptol | VC46 | alpha-Ethyl aspartate |

**Table S2.** Spearman correlation coefficient matrix (for Figure 5)

|  | **VC1** | **VC2** | **VC3** | **VC6** | **VC7** | **VC8** | **VC9** | **VC10** | **VC11** | **VC12** | **VC13** | **VC14** | **VC15** | **VC18** | **VC22** | **VC23** | **VC24** | **VC25** | **VC26** | **VC27** | **VC29** | **VC30** | **VC31** | **VC32** | **VC33** | **VC34** | **VC35** | **VC36** | **VC37** | **VC39** | **VC40** | **VC41** | **VC42** | **VC43** | **VC44** | **VC45** | **VC46** |
| --- | --- | --- | --- | --- | --- | --- | --- | --- | --- | --- | --- | --- | --- | --- | --- | --- | --- | --- | --- | --- | --- | --- | --- | --- | --- | --- | --- | --- | --- | --- | --- | --- | --- | --- | --- | --- | --- |
| ***Acetobacter*** | 0.00 | 0.50 | -0.40 | 1.00 | . | . | . | . | . | . | 0.60 | 1.00 | . | 1.00 | 1.00 | 0.74 | 0.32 | -1.00 | 0.20 | . | -1.00 | 1.00 | 0.60 | -0.50 | -0.40 | 0.90 | . | -0.30 | 0.60 | . | 0.50 | . | 0.90 | . | 0.31 | . | . |
| ***Acinetobacter*** | -1.00 | . | . | . | . | . | . | . | . | . | 1.00 | 1.00 | . | . | . | -1.00 | 0.50 | 1.00 | 0.50 | . | . | . | 0.50 | -1.00 | 1.00 | -1.00 | . | -0.50 | -1.00 | . | -1.00 | . | -1.00 | . | -0.50 | . | . |
| ***Burkholderia Paraburkholderia*** | -1.00 | . | 0.00 | . | . | . | . | . | . | . | 0.50 | 1.00 | . | . | . | -1.00 | 1.00 | -1.00 | 0.80 | . | -1.00 | . | 1.00 | -0.50 | -1.00 | 0.50 | . | -0.40 | -1.00 | . | -1.00 | . | 1.00 | . | 0.60 | . | . |
| ***unclassified_c_Cyanobacteria*** | -0.40 | -1.00 | 0.45 | -0.50 | . | . | . | . | . | . | -0.60 | -1.00 | . | 1.00 | -1.00 | -0.70 | -0.50 | 1.00 | -0.30 | . | 1.00 | -1.00 | -0.70 | 0.50 | -0.20 | -0.80 | . | 0.20 | -0.80 | . | -0.50 | . | -1.00 | . | 0.00 | . | . |
| ***Curvibacter*** | 1.00 | . | . | . | . | . | . | . | . | . | . | . | . | . | . | 1.00 | -1.00 | -1.00 | -1.00 | . | . | . | -1.00 | . | -1.00 | 1.00 | . | 1.00 | 1.00 | . | 1.00 | . | 1.00 | . | 1.00 | . | . |
| ***unclassified_f_Bacteroidales*** | 1.00 | . | -1.00 | . | . | . | . | . | . | . | . | . | . | . | . | 1.00 | -1.00 | 1.00 | 1.00 | . | . | . | -1.00 | . | 1.00 | 1.00 | . | 1.00 | 1.00 | . | 1.00 | . | -1.00 | . | 1.00 | . | . |
| ***unclassified_f_Mitochondria*** | 0.50 | -1.00 | 0.00 | -1.00 | . | . | . | . | . | . | -0.50 | -1.00 | . | . | . | -0.50 | -0.40 | 1.00 | -0.30 | . | 1.00 | . | -0.70 | 0.50 | 1.00 | -0.80 | . | 0.30 | -0.50 | . | -0.50 | . | -1.00 | . | -0.80 | . | . |
| ***unclassified_f_Oxalobacteraceae*** | 0.50 | -1.00 | -0.90 | -1.00 | . | . | . | . | . | . | -0.50 | 0.50 | . | . | . | -0.50 | 0.50 | 1.00 | 0.60 | . | -1.00 | . | 0.10 | -1.00 | 1.00 | -0.80 | . | -0.50 | -0.50 | . | -0.50 | . | -1.00 | . | -0.60 | . | . |
| ***Frateuria*** | 0.00 | -0.50 | -0.40 | -1.00 | . | . | . | . | . | . | -0.20 | 0.50 | . | -1.00 | -1.00 | -0.70 | 0.55 | 1.00 | 0.66 | . | -1.00 | -1.00 | 0.26 | -1.00 | 0.40 | -0.90 | . | -0.60 | -0.60 | . | -0.50 | . | -0.90 | . | -0.10 | . | . |
| ***Gluconobacter*** | 0.40 | -0.50 | -0.40 | -0.50 | . | . | . | . | . | . | -0.40 | 0.50 | . | -1.00 | 1.00 | -0.10 | 0.55 | 0.50 | 0.43 | . | -1.00 | 1.00 | 0.37 | -1.00 | 0.80 | -0.70 | . | -0.70 | 0.20 | . | -1.00 | . | -0.30 | . | -0.60 | . | . |
| ***Lactobacillus*** | 0.60 | 1.00 | -0.90 | -1.00 | . | . | . | . | . | . | 0.60 | 1.00 | . | -1.00 | -1.00 | -0.20 | 0.75 | 1.00 | 0.89 | . | -1.00 | -1.00 | 0.77 | -0.50 | 0.80 | 0.30 | . | 0.09 | 0.00 | . | -0.50 | . | 0.30 | . | 0.14 | . | . |
| ***Pseudomonas*** | -1.00 | . | . | 1.00 | . | . | . | . | . | . | . | . | . | . | . | 1.00 | -1.00 | -1.00 | -1.00 | . | . | . | -1.00 | . | -1.00 | 1.00 | . | -1.00 | 1.00 | . | 1.00 | . | 1.00 | . | 1.00 | . | . |
| ***Tatumella*** | -0.80 | -1.00 | 0.89 | -0.50 | . | . | . | . | . | . | -0.60 | -1.00 | . | 1.00 | -1.00 | -0.90 | -0.40 | 0.50 | -0.40 | . | 1.00 | -1.00 | -0.60 | 0.50 | -0.40 | -0.90 | . | 0.03 | -1.00 | . | -1.00 | . | -0.90 | . | -0.10 | . | . |
| ***Bacteria_others*** | 0.40 | -0.50 | 0.00 | 1.00 | . | . | . | . | . | . | -0.80 | -1.00 | . | 1.00 | 1.00 | 0.95 | -0.98 | -0.50 | -0.90 | . | 1.00 | 1.00 | -0.90 | 0.50 | -0.20 | 0.40 | . | 0.31 | 0.80 | . | 1.00 | . | 0.10 | . | 0.00 | . | . |
| ***Colletotrichum*** | . | . | . | . | . | . | . | . | . | . | . | . | . | . | . | . | . | . | . | . | . | . | . | . | . | . | . | . | . | . | . | . | . | . | . | . | . |
| ***Hanseniaspora*** | . | . | . | -1.00 | -1.00 | . | -0.10 | 1.00 | . | 1.00 | 0.50 | . | 0.32 | . | . | . | -0.80 | . | -0.60 | -1.00 | . | . | . | . | . | -1.00 | -1.00 | -0.90 | . | 0.21 | . | . | 0.30 | -0.60 | -0.80 | -0.80 | -1.00 |
| ***Penicillium*** | . | . | . | -1.00 | . | . | -0.40 | 1.00 | . | 1.00 | 0.50 | . | 0.50 | . | . | . | -1.00 | . | -0.60 | . | . | . | . | . | . | -1.00 | . | -0.80 | . | -0.60 | . | . | 0.80 | -0.40 | -0.60 | -0.80 | -1.00 |
| ***Pichia*** | . | . | . | 0.20 | 1.00 | . | 0.30 | -0.50 | . | -0.80 | 0.50 | . | 0.32 | . | . | . | -0.40 | . | 0.20 | 1.00 | . | . | . | . | . | 0.60 | 1.00 | 0.70 | . | -0.40 | . | . | -0.10 | -0.20 | 0.40 | 0.40 | 0.50 |
| ***Saccharomyces*** | . | . | . | 0.40 | 1.00 | . | 0.50 | 0.50 | . | -0.40 | -0.50 | . | -0.60 | . | . | . | 0.80 | . | 0.60 | -1.00 | . | . | . | . | . | 0.70 | 1.00 | 0.60 | . | 0.36 | . | . | -0.80 | 0.50 | 0.50 | 0.80 | 1.00 |
| ***unclassified_f_Mycosphaerellaceae*** | . | . | . | . | . | . | -1.00 | . | . | . | . | . | -1.00 | . | . | . | . | . | 1.00 | . | . | . | . | . | . | -1.00 | . | -1.00 | . | -1.00 | . | . | 1.00 | 1.00 | 1.00 | -1.00 | -1.00 |
| ***unclassified_o_Saccharomycetales*** | . | . | . | -0.20 | 1.00 | . | -0.10 | -1.00 | . | -0.80 | -1.00 | . | 0.63 | . | . | . | -0.40 | . | -0.60 | 1.00 | . | . | . | . | . | 0.20 | 1.00 | 0.00 | . | 0.15 | . | . | 0.30 | -0.10 | -0.30 | 0.60 | -0.50 |
| ***unclassified_k_Fungi*** | . | . | . | -1.00 | -1.00 | . | -0.10 | 1.00 | . | 1.00 | 0.50 | . | 0.32 | . | . | . | -0.80 | . | -0.60 | -1.00 | . | . | . | . | . | -1.00 | -1.00 | -0.90 | . | 0.21 | . | . | 0.30 | -0.60 | -0.80 | -0.80 | -1.00 |
| ***unclassified_o_Saccharomycetales*** | . | . | . | -0.50 | -1.00 | . | -0.80 | -1.00 | . | 0.50 | -1.00 | . | 0.00 | . | . | . | -0.50 | . | -0.80 | 1.00 | . | . | . | . | . | -0.80 | -1.00 | -0.80 | . | 0.32 | . | . | 0.80 | 0.00 | -0.60 | -0.50 | -1.00 |
| ***unclassified_p_Ascomycota*** | . | . | . | . | . | . | . | . | . | . | . | . | . | . | . | . | . | . | . | . | . | . | . | . | . | . | . | . | . | . | . | . | . | . | . | . | . |
| ***Zasmidium*** | . | . | . | 1.00 | . | . | -0.50 | . | . | 1.00 | -1.00 | . | -1.00 | . | . | . | 1.00 | . | 0.50 | . | . | . | . | . | . | -0.50 | . | -0.50 | . | -0.90 | . | . | 0.50 | 1.00 | 0.50 | -1.00 | -0.50 |
| ***Fungi_others*** | . | . | . | -0.50 | -1.00 | . | -0.40 | 1.00 | . | 1.00 | 1.00 | . | -0.30 | . | . | . | 0.50 | . | -0.40 | . | . | . | . | . | . | -0.80 | -1.00 | -0.80 | . | 0.40 | . | . | 0.20 | 0.00 | -0.40 | -0.50 | -1.00 |
